# The Emotion Probe: On the Universality of Cross-Linguistic and Cross-Gender Speech Emotion Recognition via Machine Learning

**DOI:** 10.3390/s22072461

**Published:** 2022-03-23

**Authors:** Giovanni Costantini, Emilia Parada-Cabaleiro, Daniele Casali, Valerio Cesarini

**Affiliations:** 1 Department of Electronic Engineering, University of Rome Tor Vergata, 00133 Rome, Italy; costantini@uniroma2.it (G.C.); daniele.casali@uniroma2.it (D.C.); 2 Institute of Computational Perception, Johannes Kepler University, 4040 Linz, Austria; emilia.parada-cabaleiro@jku.at

**Keywords:** speech, emotion recognition, artificial intelligence, English, cross-linguistic, cross-gender, SVM, machine learning, SER

## Abstract

Machine Learning (ML) algorithms within a human–computer framework are the leading force in speech emotion recognition (SER). However, few studies explore cross-corpora aspects of SER; this work aims to explore the feasibility and characteristics of a cross-linguistic, cross-gender SER. Three ML classifiers (SVM, Naïve Bayes and MLP) are applied to acoustic features, obtained through a procedure based on Kononenko’s discretization and correlation-based feature selection. The system encompasses five emotions (disgust, fear, happiness, anger and sadness), using the Emofilm database, comprised of short clips of English movies and the respective Italian and Spanish dubbed versions, for a total of 1115 annotated utterances. The results see MLP as the most effective classifier, with accuracies higher than 90% for single-language approaches, while the cross-language classifier still yields accuracies higher than 80%. The results show cross-gender tasks to be more difficult than those involving two languages, suggesting greater differences between emotions expressed by male versus female subjects than between different languages. Four feature domains, namely, RASTA, F0, MFCC and spectral energy, are algorithmically assessed as the most effective, refining existing literature and approaches based on standard sets. To our knowledge, this is one of the first studies encompassing cross-gender and cross-linguistic assessments on SER.

## 1. Introduction

With the increasing role of spoken language interfaces in human–computer interaction applications, the automatic recognition of emotional states and their modeling is of ever-growing interest [1,2,3,4,5,6,7], with several systems and datasets for speech emotion recognition (SER) being proposed by different researchers. This paper mainly faces the task of SER, with specific attention on its cross-linguistic and cross-gender implications, exploring and corroborating that on existing state-of-the-art solutions. Currently, there is no conclusive methodology for SER, or a set of results upon which all commentators can agree, mainly due to wide-ranging definitions of emotions and models for their categorization, and also due to the complex multi-dimensional nature of the task [8]. The examination of the acoustic correlates of emotional speech often relies on spurious data and collection methods that have to rely on actors or broadcast sources, with practices, such as Mood Induction Procedures (MIP) [9], being proposed to collect emotional speech data [10,11,12,13]. Along with the reproduction of “natural” emotional speech, an annotation procedure is often necessary to objectively assess the presence of a certain emotion in an utterance.

Translating “fluid” cognitive states into discrete logical categories is imperative; in this regard, there is a huge logical distinction between “Dimensional” and “Categorical” models for labeling and classifying emotions. Dimensional models, an example of which is Russell’s “circumplex” model [14], aim to identify a number of continuous dimensions, which can characterize emotions in a spectrum-like manner. On the other hand, categorical models simply see each emotion as a different “class”, with no immediate qualitative common ground.

A common automatic Machine Learning (ML) -based framework of an SER system could be roughly summarized with five main modules: speech input, extraction of acoustic or mathematical features, feature selection, classification, and emotion output. Predictably, such as framework, is roughly based on “categorical” models of emotion classification, since they rely on domain-specific features, which, in turn, lead to a multi-class problem. The basic assumption is that there is a set of objectively measurable parameters in voice that reflect the affective state a person is currently expressing. This assumption is also supported by the fact that most psychological states involve physiological reactions, which, in turn, modify the process by which voice is produced. For example, anger often produces changes in respiration and increases muscle tension, influencing the vibration of the vocal folds and vocal tract shape and affecting the acoustic characteristics of the speech [15]. More superficially, many emotions can indeed be noticed “by ear”, especially among individuals belonging to the same ethnic and social environment. Within this framework, it is logical to think of emotions as separate “classes”, in a finite number. Naturally, since emotions are a product and a dynamic of each person’s psychology, it is impossible to actually categorize them without approximation. Therefore, whereas some studies have concentrated on the identification of the greatest possible number of emotions, others have favored the division into macro-categories, only employing those considered to be the “main” emotional states: neutrality, happiness, sadness, fear and anger.

### Related Works and Datasets

A relevant issue within the field of SER has always been represented by the differences among languages, expressing emotions differently due to both cultural and phonetic reasons. Cross-language and cross-gender [16,17] studies should help pave the way for the identification of independent parameters and common dynamics that may generalize the physiological and vocal phenomena related to a certain emotion.

The most common algorithmic framework for automatic SER involves the extraction of features from audio data, in turn used to train classifiers. Despite various issues, including the scarcity of datasets or the presence of inter-individual differences, most studies involve either “traditional” ML classifiers (Support Vector Machines (SVM)) [18,19,20,21,22,23], neural networks (Multi-Layer Perceptrons (MLP)) [24], Long Short Term Memory (LSTM) networks, Deep Belief Networks (DBN) [22] or Convolutional Neural Networks (CNN) [25,26,27], and probabilistic models (Hidden Markov Models (HMM)) [6,28]. Table 1 outlines an overview of some representative works in the field of SER, along with the datasets and emotions used and classification accuracy, showing the prevalence of neural networks and SVM, often favored in other speech-based ML tasks as well [29].

Many datasets exist, and the main criticalities are the amount of data, the quality of the recording, the procedure used to induce (or detect) emotions, and the annotation process for the final labeling. Some common datasets for SER are mentioned in Table 1: EMO-DB [30], also called Berlin, is a widely-used database of German rehearsed speech, with acted emotions, recorded in a professional environment and divided into seven classes. IEMOCAP [31], on the other hand, contains approximately 12 h of rehearsed and free (pseudo-induced) emotional speech by American actors, encompassing a total of five classes: Happy, Sad, Fear, Frustration and Neutral, along with other data, regarding motion and non-verbal cues. Currently, there is no solid agreement on the validity or similarity of all the possible emotion-inducing procedures, with professional acting being often employed due to logistic reasons for the ease of recording. While detractors deem acted emotions to be forced and more exaggerated than real ones [32,33], it is also argued that professional actors can effectively auto-induce certain states on themselves, in fact making the feeling “real” [34,35], and that social context creates artifacts and limits spontaneity anyway [36].

As for cross-linguistic studies, Rajoo and Aun [37] proved the strong language-dependent nature of SER, which was further explored by Fu et al. [38], who trained algorithms with combinations of three languages, obtaining accuracies which, preliminarily, outlined the possibility of a cross-language model for German and Chinese, while Italian was not recognized as successfully—possibly due to the unbalanced dataset. Li and Akagi [39] obtained interesting results, merging widely known existing datasets, whereas Tamulevičius et al. [40] obtained high accuracies with a CNN-based approach. However, their dataset is highly unbalanced, and the emotions have been acted by non-professionals.

For the aim of this study, three widely used classifiers are employed, namely SVM, Naïve Bayes and MLP, trained on acoustic features resulting from a novel selection procedure. Despite a certain prevalence of deep and convolutional neural network-based solutions for many SER datasets of today, the need to face the problem from a broader point of view, which could also include cross-linguistic implications, and the subsequent choice of datasets, led us to choose traditional ML algorithms, which could provide reliable results for medium-to-small amounts of training data [41]. Moreover, although deep learning models do not require specific feature engineering, traditional ML algorithms rely on proper selection methods, on carefully extracted acoustic features, often in standard sets [5,42], which we tried to employ with a pipeline, involving Kononenko discretization and a correlation-based selection. The dataset chosen for our experimentation, called Emofilm [43], comprises the same emotional sentences uttered by different speakers in three languages (English, Spanish and Italian), and it is roughly balanced in gender. Other than presenting a working framework that may bring a high accuracy for SER tasks, exploring the feasibility of a cross-linguistic approach and observing the trends within emotions among cultures is one of the center features of our work.

The paper is organized as follows: The following “Materials and Methods” section will detail the dataset, the subsequent classification task, the ML framework, and briefly explain the theory and reasoning behind our algorithms of choice. Experimental results for SER on the Emofilm dataset will be presented in the “Results” section and will be discussed afterwards, along with a deeper analysis of the acoustic features.

In the present work, emotions are translated into classes, and will be interchangeably referred to using the associated noun or adjective; as an example, the “Happiness” class can also be referred to as “Happy”.

## 2. Materials and Methods

### 2.1. Dataset

Although numerous multi-language corpora have been collected already, almost none of these is configured on a full cross-linguistic information basis, which led to us choosing Emofilm [43]. Emofilm is a cross-linguistic SER database comprising 1115 audio sequences extracted from 43 movies, for a grand total of 207 speakers (94 females and 113 males). It encompasses five emotions, namely: Happy, Sad, Fear, Anger and Disgust. It is divided into three languages, with English being the original language of the movies, and Spanish and Italian being dubbed versions. Dubbing is common practice in Spain and Italy, with dubbers being trained as actors and voice professionals; the dynamics of the original movie are thus professionally re-enacted, along with the emotions that come with them [47,48].

Each movie has produced a number of emotional clips, which have then been annotated [43] in order to assess the emotional content for each language. Therefore, there are 413 clips by Italian speakers (190 of them spoken by females and 223 by males), 360 clips by English speakers (182 of them spoken by females) and 342 clips by Spanish speakers (165 of them spoken by females). Of the 94 female speakers, 35 spoke English, 35 Italian and 24 Spanish. Of the 113 male speakers, 44 spoke English, 36 Italian and 33 Spanish. The correspondence is not perfect, which means that sometimes the same dubber has dubbed more than one English movie. We anticipate that male, female and the three languages will be classified both within unified subsets and separately. The clip selection was created manually, prioritizing realistic performances and rejecting clips that were unintelligible or affected by background noise and/or music. As a result, all audio files were already trimmed and noise-free. Audio was extracted for each clip as a separate mono file with a 48 kHz sample rate and a 16-bit depth in PCM-wave format.

The reasons behind the choice of Emofilm can be summarized as follows:

1. Emofilm contains clips made of the very same sentences uttered in three languages, and is therefore homogeneous in terms of context and acted emotions;
2. The three languages encompassed by Emofilm are all of European origin and belong to Western culture;
3. Actors and dubbers are trained professionals, ensuring the best possible performance on acted emotions;
4. The voice is professionally recorded and processed.

**Figure 1:**
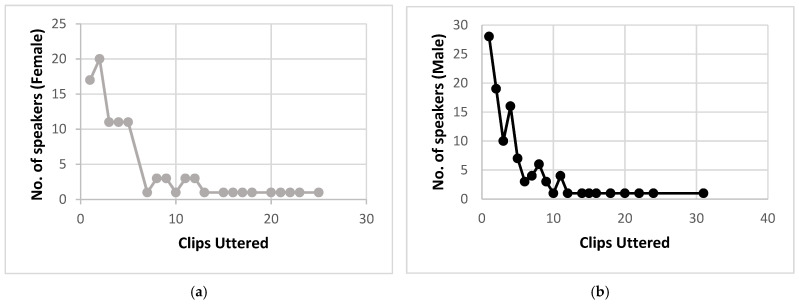
Number of speakers that uttered a certain number of clips (**a**) Female; (**b**) Male. As an example, 17 females uttered one clip, 20 females uttered 2 clips and, finally, one female uttered 25 clips (last point on the *x*-axis).

Table 2 depicts the distribution of clips for each emotion, divided by language and gender. It shows that the Emofilm dataset is relatively balanced, with the “Happiness” class being slightly under-represented. The five emotions encompassed are the “basic” emotions in SER, plus Disgust which is a very common emotion in movies and, most importantly, bears relevant differences against the other four. Many speakers uttered more than one clip: the distribution for males and females can be found in Figure 1. The following abbreviations will be used from now on: “F” and “M” for female and male respectively, and “It”, “Sp” and “En” for Italian, Spanish and English languages respectively. Emotion labels are abbreviated as such: “DIS” = Disgust; “HAP” = Happy; “FEAR” = Fear; “ANG” = Angry; “SAD” = Sad.

### 2.2. Machine Learning Framework

The framework for the preparation and classification of audio data is organized as such:

1. Feature Extraction followed by a standardization procedure;
2. Discretization, using Kononenko’s criterion [49];
3. Feature Selection, using a Correlation-based Feature Selector (CFS);
4. Training of Classifiers, namely SVM, Naïve Bayes (NB) and Multi-layer Perceptron (MLP). The three classifiers are independently trained on the same feature sets;
5. Emotion Output, which in our specific case comes out of a 10-fold cross-validation;
6. Statistical analysis of the obtained results.

Steps 2 to 5 have been implemented using Weka [50] by the University of Waikato.

**Figure 2:**
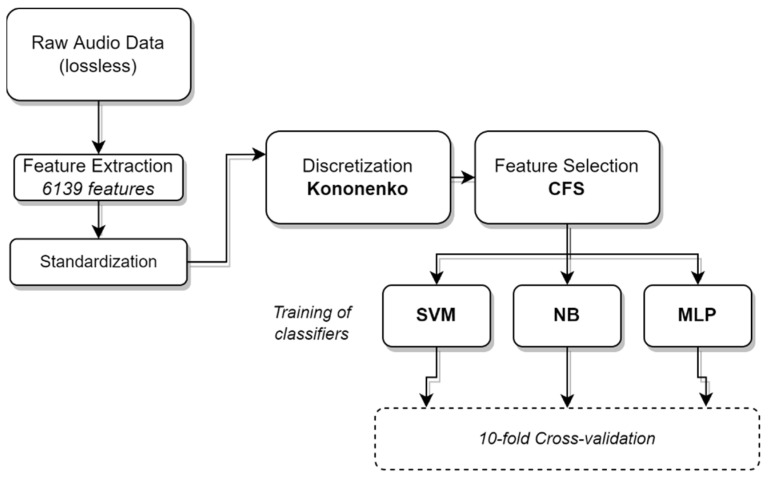
Flowchart for the SER Machine Learning framework.

Figure 2 outlines the system.

### 2.3. Feature Extraction

A set of acoustic features needs to be determined for every SER application. Although many sets have been proposed and many studies agree on using specific domains, namely energy, pitch, prosody and cepstrum [51], the cross-linguistic nature of this study and the need for generalization called for a wide, non-standard set of features to then be reduced. The feature set of choice comes from the INTERSPEECH 2013 library [52], embedded in the feature extraction tool OpenSMILE (by Audeering) [53]. A total of 6139 features are extracted, each one actually being a static functional applied on low-level signal descriptors (LLDs) from basic speech features (pitch, loudness, voice quality) or representations of the speech signal (cepstrum, linear predictive coding). The functionals that are applied are: extremes (position of max/min value), statistical moments (first to fourth), percentiles (ex. the first quartile), duration (e.g., percentage of time the signal is above threshold) and regression (e.g., the offset of a linear approximation of the contour). Choosing a predefined set would have been in contrast with our aim to investigate the widest number of features and domains to then isolate the most relevant. Moreover, OpenSMILE provides high-level features with windows already unified by means of smoothed moving average. After extraction, the feature vectors are standardized so the distribution of the values of each feature has a mean equal to 0 and a standard deviation of 1.

### 2.4. Kononenko’s Discretization

As a first simplification, discretization has been applied on all features. Although we did experiment with applying it after the feature selection and before classification, better results were obtained when the whole feature set was already discretized.

The algorithm of choice is Kononenko’s criterion [49], which is a recursive algorithm using the Minimum Description Length principle (MDL) [54,55] as a stopping criterion. It is based on a non-myopic take of Kira and Rendell’s RELIEF algorithm [56], optimized for being able to deal with multi-class problems, robust to noise and acceptably unbiased. For each attribute, the algorithm proceeds considering two near instances which may generate a “hit” (H) if they belong to the same class and have the same range of the attribute, or a “miss” if they have the same range of the attribute but belong to different classes. The formula for the weight W of each attribute is:

W(att) = P_H_ − P_M_ (1)

where P_H_ and P_M_ are probabilities of hit and miss respectively.

The algorithm aims to find the best set of boundaries and is embedded in a greedy discretization procedure [57] stopping when the chosen heuristic, based on MDL, is worse than the previous step.

### 2.5. CFS: Correlation-Based Feature Selection

After being discretized, the whole feature set endured a selection using Hall’s correlation-based criterion (CFS) [50,58]. The CFS computes merit factors for subsets of features, the best of which has been selected by a Best First search method. The formula for the merit factor is:

(2) $$M_{s}=\frac{{k\;r}_{\mathrm{cf}}}{\sqrt{k+k\left(k-1\right)r_{\mathrm{ff}}}}$$

where k is the number of features in the subset, r_cf_ is the average correlation between each feature and the class, and r_ff_ is the average cross-feature correlation.

The main principle is to select the features that hold the greatest separation potential, while also removing redundancy.

The search method retains a non-homogeneous number of features, usually around 1–3% of the original, so each final classification task will be based on a different number of features.

### 2.6. Classification

Three classifiers, namely SVM, NB and MLP, have been independently trained for each task. The classifiers have been chosen in order to investigate state-of-the-art algorithms, such as SVM and neural networks, including MLP. Naïve Bayes, on the other hand, has been employed as a means of comparison. No deep or convolutional networks have been employed, since our focus was mainly on acoustic features-based algorithms; moreover, the small amount of training data also called for the usage of “traditional” ML algorithms.

#### 2.6.1. SVM (Support Vector Machine)

As outlined in the Introduction, SVM’s are widely used non-probabilistic classifiers, especially common with medium-to-small datasets due to their generalization power. A general SVM is a binary classifier based on finding the optimal separation hyperplane between the two nearest examples of opposite classes, called “support vectors” [19]. The problem is solved with the Lagrange dual formula with “soft-margins” thanks to the introduction of the parameter C, which is the “Complexity” of penalizing classification errors during training.

We used a soft-margins linear SVM, solved with Platt’s SMO algorithm and adapted to a multi-class scenario with a tree of one-vs-one comparisons.

#### 2.6.2. NB (Naïve Bayes)

Naïve Bayes is a probabilistic classifier based on Bayes’ theorem which operates under the assumption that attributes are independent from each other—hence the name “Naïve” [59]. The algorithm takes advantage of the Bayes’ Theorem to compute the posterior probability distribution and has often brought interesting and generalized results for voice analysis despite its straightforward nature.

#### 2.6.3. MLP (Multi-Layer Perceptron)

The Multi-Layer Perceptron is the “basic” example of a Neural Network. It is an algorithm based on layers of fully-connected combination blocks with non-linear activation functions in between, usually ending with a softmax layer and a threshold-based classifier. The basic training mechanism involves back-propagation of the error, triggering the update of the weights [60].

In our case, a number of hidden layers equal to half the number of attributes + number of classes—one has been employed for each classification task, and a sigmoid activation function has been used.

## 3. Results

### 3.1. Classification Tasks

The Emofilm dataset comprehends speech from female and male actors in three languages, divided into clips. All possible individual, cross-language and cross-gender classifications for SER have been explored. The following classification tasks have been organized:

1. Monolingual with gender variations: a single language (It, Sp, En) with males only (M), females only (F), or both (M + F).
2. Bilingual without gender variations: two languages (It + Sp, It + En, Sp + En) with both genders (M + F); these couplings aim to explore whether there were more poignant similarities between any two out of three languages; for this reason, no cross gender comparison has been considered for these tasks.
3. Multilingual (All) with gender variation: all languages (It + Sp + En) with only M, only F, or M + F; this aims to obtain a single SER tool.

### 3.2. Experimental Results

**Figure 3:**
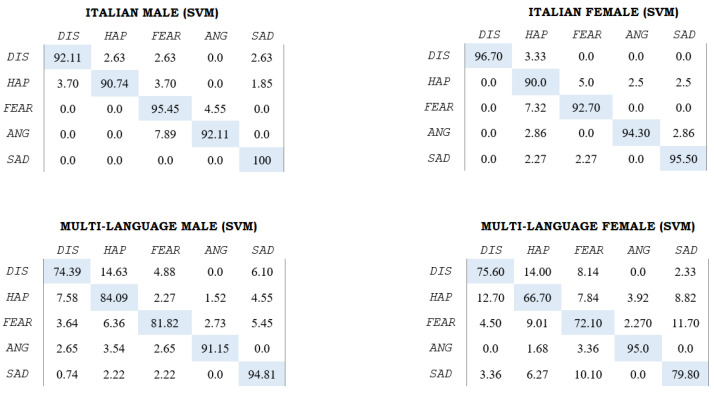
Confusion matrices for the SVM classifier for the It M, It F, All M and All F comparisons. Emotion labels are thus abbreviated: “DIS” = Disgust; “HAP” = Happy; “FEAR” = Fear; “ANG” = Angry; “SAD” = Sad.

Since three classifiers have been employed for each comparison, an overview outlining weighted accuracies (WA) for each task, number of features and best and worst emotions, in terms of accuracy for the sole MLP classifier, is reported in Table 3. For additional information, confusion matrices for the sole SVM classifier, for the It M, It F and All (M and F) comparisons, are reported in Figure 3. The WA is weighted according to the class distribution, which can be found in Table 2, taking into account the slight imbalances between emotions. As a practical example, taking a look at the It M SVM matrix, the accuracy value of 92.1% for the “dis” class is weighted with a factor of 37, and the total is then divided by 165.

Let the reader be reminded that each per-class accuracy is the result of a 10-fold cross-validation; therefore, it is already the average of 10 values.

### 3.3. Statistical Analysis

Before discussing the trends observed in Table 3, we performed a statistical assessment to verify the significance of the differences between accuracies. Firstly, in order to compare the three different algorithms (NB, SVM and MLP), we performed a Wilcoxon signed-rank test [61] on each combined pair over the WA columns. We chose this test because the considered values cannot easily be assumed to be normally distributed [62]. The results of the Wilcoxon test in terms of *p*-values are:

1. NB vs. SVM: *p* = 0.001609
2. NB vs. MLP: *p* = 0.0001964
3. MLP vs. SVM: *p* = 0.001474

A commonly considered significance level for this test is a *p*-value less than 0.05, which means that the differences between the three classifiers can be considered statistically relevant. However, it is most evident with NB versus MLP.

We also performed a statistical assessment on the differences between single versus cross-gender tasks and single versus double-language. In this case, since the distribution can be assumed to resemble a Gaussian, we employed a Student t-test, with 17 degrees of freedom [63]. The results are:

1. Single-gender vs. cross-gender: *p* = 1.092 × 10^−13^
2. Single-language vs. double-language: *p* = 1.285 × 10^−8^

Low *p*-values show that differences are significant in both cases, with the single versus cross-gender dynamics bearing a much lower *p*-value and, thus, an even more significant difference.

## 4. Discussion

As shown in Table 3, all the classifiers obtained high accuracy values for a five-way classification task, with the MLP always bringing the highest, shortly followed by SVM. NB obtains slightly lower accuracies in almost all cases, and the differences between MLP and NB are the most statistically significant.

Since Neural Networks and SVM have very different dynamics, a preliminary conclusion could be that both are relevant solutions in SER, as already noted in the literature.

Predictably, accuracy drops when considering both genders or more than one language, which is in line with the more complex nature of cross-language and cross-gender SER tasks. Especially looking at dual language tasks, it appears that merging two genders (M + F) has greater effects on the overall accuracy than merging languages, which is also validated by the Student t-test. This suggests that the differences in expressing emotions, or reflecting them through speech, between male and female subjects, is more relevant than cross-linguistic differences, at least between two similar cultures. As far as we know, this is one of the first studies exploring these phenomena and preliminarily observing this.

The dual language tasks show higher accuracies for Italian merged with Spanish, which could be in line with the inherent similarities between the two languages and cultures, while Italian merged with English obtains the lowest accuracies.

Predictably, the multi-language approach holds the lowest accuracies, with a lower, but acceptable, 67.26% for the cross-gender version.

Considering accuracies on the single class/emotion, there is an evident trend of higher accuracies for emotions with negative valence, namely Sadness and Anger. Although this could suggest a cross-linguistic tendency to express negative emotions in a clearer way, it could also be linked to the fact that actors purposefully exaggerate these emotions, and could be considered a limitation of the dataset and of the methodology itself. Although accuracies are relatively similar throughout the five classes, MLP and SVM, which we assessed as being the best performing classifiers, do show slightly different trends. As an example, for the It M comparison, the least accurate emotion for the SVM classifier is Happy, whereas for the MLP it is Disgust. All MLP classifiers but one assessed the Happy emotion as the most difficult to detect for female speakers, which could derive from imbalances in acting performances, but could suggest a tendency for females to better express low-arousal emotions [64].

### 4.1. Acoustic Features Analysis

Most of the existing approaches for SER rely on standard sets of features, often encompassing domains, such as pitch, jitter or MFCC (Mel-Frequency Cepstral Coefficients [65]). Our procedures algorithmically selected a number of features, all taken from the INTERSPEECH 2013 set of features for speech signals. In order to observe trends in our selected features, we first assessed eventual similarities between couples of training sets, cross-checking the position of every feature, to see if we could find a feature (or more) present in both sets. In fact, it is impossible to define a group of features that are definitely linked with a given language or gender in our study. The same behavior can be observed for both genders.

This indicates that, although cross-gender and cross-linguistic SER tasks are indeed feasible, there is no clear proof of a “universal” set of features [66], at least from an LLD point of view. However, there is a definite trend for feature domains, recurrent throughout all classification tasks, actually making up around 88% of the full feature sets (averaged by each task). The four domains are RASTA-PLP filtering [67], F0 or fundamental frequency extracted, using Hermes’ subharmonic summation algorithm (SHS) [68], MFCC and spectral energy. While the last three have somehow been often used in the literature, the usage of RASTA is still seminal and underrated. However, RASTA is a frequency-of-frequency kind of filtering, based on an all-pole model, which is inherently noise-robust and insensitive to slowly varying spectral components, and often improves classification performances for speech tasks with respect to similar domains [69,70,71,72].

As a final validation, we removed all features not pertaining the four above-mentioned domains and re-evaluated the classification accuracy for all single-language, single-gender tasks. We observed a maximum decrease in final accuracy of 2.1%, which further suggests that RASTA, F0, spectral energy and MFCC may be a good starting point and a reasonably comprehensive feature set to use in SER. An example of a feature list for the It M task, with names from OpenSMILE, can be found in Appendix A. For this specific task, the removal of all features not belonging to the four domains brings a WA of 93.3% (SVM classifier), which is 0.9% less than the original one. The complete list of features for all tasks can be found in the Supplementary Materials.

### 4.2. Limitations

This study focuses on verifying the feasibility of cross-language, cross-gender SER and exploring related dynamics. We, accordingly, chose a dataset that offers well-recorded, professionally acted emotional clips, in three Western languages, within the very same context. The dataset itself presents some inherent limitation: other than its slight imbalances, its size of roughly 400 instances per language could be considered small, especially compared to other existing datasets. Moreover, even accepting the concept of self-induction for professionally acted emotions, some exaggeration or artifacts could affect the classification results, as noted for the predominance of “negative” emotions among the most accurate ones. Imbalances in the number of emotional clips uttered by each subject can also influence the results. Although most speakers either uttered clips for many emotions (usually 3 to 5), or only uttered one single clip, artifacts due to peculiar training-validation splits could occur.

Another inherent drawback of this study is the limited number of emotions, which is a never-ending issue in SER per se, and especially the usage of only three languages. However, being already conscious of the profound inter-cultural differences among languages, and of the complexity of the problem itself, we chose to focus on Western languages, based on a similar cultural background. Expanding our work to encompass more languages, especially non-Western ones, is definitely one of our aims for the future. Many widely used datasets exist for such languages, such as CASIA, but a cross-corpus analysis would definitely add another challenge to our work. We are currently experimenting the presented methodologies and feature domains on the EMODB dataset. On the other hand, although it would also be interesting to expand the Emofilm dataset with clips dubbed in other languages, the proficiency of the dubbers, as well as the tendency of some nations to just provide subtitles, need to be taken into account.

As for methodology, we consciously concentrated on finding generalized feature domains and employing “traditional” ML algorithms, without relying on deep learning. However, the possibilities of such an approach are undeniable and must be considered when tackling such a complex task.

## 5. Conclusions

We evaluated the performances of three classifiers (SVM, NB, MLP) on the Emofilm dataset, which contains data from male and female subjects in three languages. The main objective was to propose and validate the ML framework and a feature selection procedure based on Kononenko’s discretization, followed by a CFS, and to explore the feasibility and the dynamics of cross-gender and cross-linguistic SER. Experimental results yield high accuracies and prove the feasibility of a multi-language SER; mean weighted accuracies for single-language tasks are 95.5% and 83.6% for single and dual-gender configurations, respectively; dual-language single-gender tasks yield a mean accuracy of 87.9%; three-language tasks brought 82.8% and 67.3% accuracies for single and double-gender configurations. To our knowledge, this is the first study comprehensively employing the Emofilm database, thus, obtaining state-of-the-art results on it. The accuracy drops between tasks preliminarily suggest that merging male and female subjects within the same language results in a harder SER than merging two languages. Thus, the differences between male and female in expressing emotions are assessed as crucially relevant, possibly even more than cultural and phonetic differences between languages belonging to a similar cultural background. These considerations are backed by statistical analysis. The domains of RASTA, F0, MFCC and spectral energy are assessed as generally effective for SER, stressing the potential of RASTA filtering. Nevertheless, no universal set of specific feature descriptors could be established for each task, which suggests that emotions through languages and gender can indeed be identified, but require slightly different features to be considered. To our knowledge, this is one of the first studies encompassing a cross-linguistic approach for SER with success, using languages from Western culture countries. On the other hand, it would definitely be beneficial to expand the study to non-Western cultures and non-European languages to identify culture-specific features, eventually also establishing a global set of feature domains.

## Figures and Tables

**Table 1:** Review of the most representative works in SER and the classifiers they employed.

Study	Year	Database	Emotions	Features	Classifier	Reported Results
Alonso et al. [21]	2015	EMO-DB [30], others	Happy, Angry, Sad, Bored.	Spectral, Prosody, Pitch	SVM	94.9% (EMO-DB)
Shukla et al. [28]	2016	SUSAS [44]	Neutral, Angry, Sad, Lombard, others.	MFCC	HMM	93.9%
Wen et al. [22]	2017	EMO-DB, SAVEE [32], CASIA	Neutral, Happy, Angry, Sad, Fear, Disgust, Surprise.	Spectral, Prosody, Hu Moments	DBN, SVM	82.3% (EMO-DB) 53.6% (SAVEE) 48.5% (CASIA)
Sun et al. [23]	2019	EMO-DB, CASIA	Neutral, Happy, Angry, Sad, Bored, Fear, Disgust.	Spectral, Prosody, MFCC, Voice Quality	SVM	86.7% (EMO-DB) 83.7% (CASIA)
Kerkeni et al. [45]	2019	EMO-DB, Spanish	Neutral, Happy, Angry, Sad, Bored, Fear, Disgust.	MFCC, Spectral	SVM, RNN	83% (EMO-DB) 94% (Spanish)
Aftab et al. [26]	2021	EMO-DB, IEMOCAP [31]	Neutral, Happy, Angry, Sad, Bored, Fear, Disgust.	-	CNN	94.2% (EMO-DB) 79.9% (IEMOCAP)
Zehra et al. [46]	2021	EMO-DB, SAVEE, EMOVO [34]	Neutral, Happy, Angry, Sad, Fear, Disgust, Surprise.	MFCC, Spectral, Prosody	SVM	Many (single and cross-corpora)
Gat et al. [27]	2022	IEMOCAP	Neutral, Happy, Sad, Angry	-	Gradient-base Adversary Learning	81%

**Table 2:** Number of clips for each emotion, each language and each gender. As an example, “It M” means “Italian Males” and there are 37 clips of Italian males labelled with the “Disgust” emotion. Last column/row shows the total of clips for all emotions/tasks.

Task	Dis	Hap	Fea	Ang	Sad	Total
It M	37	23	33	41	31	165
It F	35	27	37	36	43	178
Sp M	33	33	29	43	44	182
Sp F	31	17	47	39	43	177
En M	41	30	40	35	44	190
En F	44	38	54	38	49	223
Total	221	168	240	232	254	

**Table 3:** Classification performances in terms of weighted accuracy. Emotion labels are thus abbreviated: “DIS” = Disgust; “HAP” = Happy; “FEAR” = Fear; “ANG” = Angry; “SAD” = Sad. “Best” and “Worst emotion” refer to the most and least accurate classes.

Classification: Language(s)	Classification: Gender(s)	No. of Features	WA (%): SVM	WA (%): NB	WA (%): MLP	Best Emotion	Worst Emotion
It	M	160	94.2	89.7	96.0	sad	dis
It	F	177	93.7	89.5	94.2	sad	hap
It	M + F	176	80.4	77.0	83.3	ang	fea
Sp	M	158	97.2	95.5	97.7	sad	dis
Sp	F	163	91.8	91.2	91.8	ang	hap
Sp	M + F	167	82.5	82.5	85.0	sad	dis
En	M	166	97.2	95.5	97.2	sad	fea
En	F	173	94.6	94.6	95.8	sad	hap
En	M + F	149	81.9	78.4	82.5	ang	hap
It + Sp	M	196	89.8	84.3	91.0	ang	dis
It + Sp	F	202	85.2	84.0	88.4	ang	fea
Sp + En	M	199	89.0	85.1	89.9	ang	dis
Sp + En	F	176	84.4	85.1	89.9	ang	hap
It + En	M	215	85.8	80.6	85.8	sad	dis
It + En	F	185	79.7	81.4	82.5	ang	hap
All	M	215	85.3	77.7	85.3	sad	fea
All	F	195	78.4	76.4	80.3	sad	hap
All	M + F	204	67.3	60.6	67.3	ang	fea

## Data Availability

Restrictions apply to the availability of these data. Data were obtained from the Emofilm database and are available at https://zenodo.org/record/1326428 (accessed on 19 February 2022).

## Supplementary Materials

The following supporting information can be downloaded at: https://www.mdpi.com/article/10.3390/s22072461/s1, File S1: Full feature list (featurenames.xlsx).

## Appendix A

The following table outlines the feature list for the It M classification task. The features are not ranked in any particular order, and the name is the original one found in the INTERSPEECH configuration file [52] of OpenSMILE. Removal of all features that do not start with the prefixes “audspec”, “pcm_Mag”, “F0final”, “mfcc” results in a negligible loss of classification accuracy. In this specific case, the features that would be removed are related to jitter, shimmer and zero-crossing rate.

**Table A1:** Feature list for the It M classification task. Please note that numbers in square brackets are not references, but refer to the number of the window for the specific filtering.

It M—Feature List
audSpec_Rfilt_sma[7]_leftctime
audSpec_Rfilt_sma[8]_quartile1
audSpec_Rfilt_sma[10]_quartile1
audSpec_Rfilt_sma[10]_lpc4
audSpec_Rfilt_sma[11]_leftctime
audSpec_Rfilt_sma[12]_lpc4
audSpec_Rfilt_sma[15]_lpc3
audSpec_Rfilt_sma[16]_maxPos
audSpec_Rfilt_sma[20]_risetime
audSpec_Rfilt_sma[21]_minPos
audSpec_Rfilt_sma[22]_percentile1.0
audSpec_Rfilt_sma[25]_percentile1.0
pcm_Mag_fband250-650_sma_lpgain
pcm_Mag_fband250-650_sma_lpc0
pcm_Mag_fband250-650_sma_lpc2
pcm_Mag_fband1000-22000_sma_iqr2-3
pcm_Mag_fband1000-22000_sma_lpc3
pcm_Mag_spectralRollOff50.0_sma_quartile2
pcm_Mag_spectralRollOff75.0_sma_quartile1
pcm_Mag_spectralRollOff75.0_sma_risetime
pcm_Mag_spectralRollOff90.0_sma_risetime
pcm_Mag_spectralFlux_sma_lpc0
pcm_Mag_spectralCentroid_sma_quartile1
pcm_Mag_spectralCentroid_sma_lpc1
pcm_Mag_spectralEntropy_sma_lpc0
pcm_Mag_spectralVariance_sma_quartile3
pcm_Mag_spectralVariance_sma_iqr1-3
pcm_Mag_spectralKurtosis_sma_quartile1
pcm_Mag_harmonicity_sma_quartile2
mfcc_sma[1]_quartile1
mfcc_sma[1]_quartile3
mfcc_sma[1]_pctlrange0-1
mfcc_sma[1]_skewness
mfcc_sma[1]_leftctime
mfcc_sma[2]_percentile1.0
mfcc_sma[2]_lpc0
mfcc_sma[3]_quartile1
mfcc_sma[4]_skewness
mfcc_sma[6]_maxPos
mfcc_sma[6]_percentile1.0
mfcc_sma[6]_upleveltime75
mfcc_sma[6]_lpc1
mfcc_sma[8]_lpgain
mfcc_sma[10]_maxPos
mfcc_sma[10]_quartile2
mfcc_sma[10]_stddev
mfcc_sma[11]_quartile3
mfcc_sma[11]_percentile1.0
mfcc_sma[12]_quartile3
mfcc_sma[13]_percentile99.0
mfcc_sma[13]_upleveltime75
mfcc_sma[14]_percentile1.0
mfcc_sma[14]_skewness
mfcc_sma[14]_upleveltime50
audSpec_Rfilt_sma_de[2]_leftctime
audSpec_Rfilt_sma_de[8]_iqr1-3
audSpec_Rfilt_sma_de[13]_lpc0
audSpec_Rfilt_sma_de[21]_quartile2
audSpec_Rfilt_sma_de[23]_quartile2
audspec_lengthL1norm_sma_iqr2-3
pcm_zcr_sma_skewness
audspec_lengthL1norm_sma_de_range
audspec_lengthL1norm_sma_de_stddev
audspec_lengthL1norm_sma_de_lpc4
audspecRasta_lengthL1norm_sma_de_iqr2-3
pcm_Mag_fband250-650_sma_de_iqr1-3
pcm_Mag_fband1000-22000_sma_de_iqr1-3
pcm_Mag_spectralRollOff25.0_sma_de_minPos
pcm_Mag_spectralRollOff25.0_sma_de_percentile1.0
pcm_Mag_spectralRollOff50.0_sma_de_leftctime
pcm_Mag_spectralFlux_sma_de_iqr1-3
pcm_Mag_spectralFlux_sma_de_lpgain
pcm_Mag_spectralCentroid_sma_de_quartile2
pcm_Mag_spectralCentroid_sma_de_percentile1.0
pcm_Mag_spectralSkewness_sma_de_iqr2-3
pcm_Mag_spectralSlope_sma_de_lpc2
pcm_Mag_harmonicity_sma_de_upleveltime50
mfcc_sma_de[2]_iqr1-3
mfcc_sma_de[2]_percentile1.0
mfcc_sma_de[3]_lpgain
mfcc_sma_de[3]_lpc1
mfcc_sma_de[4]_minPos
mfcc_sma_de[4]_lpc3
mfcc_sma_de[5]_percentile1.0
mfcc_sma_de[5]_lpgain
mfcc_sma_de[6]_iqr1-2
mfcc_sma_de[6]_lpc0
mfcc_sma_de[7]_quartile2
mfcc_sma_de[11]_percentile99.0
mfcc_sma_de[13]_skewness
mfcc_sma_de[14]_leftctime
F0final_sma_rqmean
F0final_sma_quartile1
F0final_sma_quartile2
F0final_sma_quartile3
F0final_sma_skewness
F0final_sma_upleveltime25
jitterLocal_sma_linregc1
jitterLocal_sma_iqr1-2
jitterLocal_sma_iqr1-3
shimmerLocal_sma_iqr2-3
shimmerLocal_sma_iqr1-3
shimmerLocal_sma_lpc0
F0final_sma_de_qregc1
F0final_sma_de_risetime
jitterLocal_sma_de_posamean
jitterLocal_sma_de_iqr1-2
audspec_lengthL1norm_sma_qregc3
audSpec_Rfilt_sma[0]_flatness
audSpec_Rfilt_sma[5]_minRangeRel
audSpec_Rfilt_sma[6]_peakMeanAbs
audSpec_Rfilt_sma[6]_peakMeanRel
audSpec_Rfilt_sma[11]_minRangeRel
pcm_Mag_fband250-650_sma_linregc1
pcm_Mag_fband250-650_sma_qregc1
pcm_Mag_fband1000-22000_sma_peakRangeAbs
pcm_Mag_fband1000-22000_sma_qregc3
pcm_Mag_spectralRollOff25.0_sma_qregc2
pcm_Mag_spectralRollOff90.0_sma_flatness
pcm_Mag_spectralFlux_sma_stddevFallingSlope
pcm_Mag_spectralEntropy_sma_qregc3
pcm_Mag_spectralVariance_sma_meanFallingSlope
pcm_Mag_spectralSkewness_sma_peakMeanMeanDist
pcm_Mag_spectralSlope_sma_peakRangeRel
pcm_Mag_harmonicity_sma_rqmean
pcm_Mag_harmonicity_sma_peakRangeRel
pcm_Mag_harmonicity_sma_peakMeanAbs
mfcc_sma[1]_peakDistStddev
mfcc_sma[1]_peakMeanAbs
mfcc_sma[1]_meanFallingSlope
mfcc_sma[1]_qregc3
mfcc_sma[2]_linregerrQ
mfcc_sma[4]_rqmean
mfcc_sma[5]_meanFallingSlope
mfcc_sma[8]_peakMeanRel
mfcc_sma[9]_peakMeanAbs
mfcc_sma[9]_peakMeanMeanDist
mfcc_sma[10]_peakMeanRel
mfcc_sma[11]_peakMeanAbs
mfcc_sma[12]_peakDistStddev
mfcc_sma[12]_peakMeanRel
mfcc_sma[13]_stddevRisingSlope
mfcc_sma[13]_qregc2
mfcc_sma[14]_stddevFallingSlope
audspec_lengthL1norm_sma_de_posamean
audspec_lengthL1norm_sma_de_peakMeanMeanDist
audspec_lengthL1norm_sma_de_meanFallingSlope
audSpec_Rfilt_sma_de[18]_minRangeRel
audSpec_Rfilt_sma_de[24]_peakMeanRel
audSpec_Rfilt_sma_de[25]_peakRangeRel
pcm_Mag_fband1000-22000_sma_de_peakMeanAbs
pcm_Mag_spectralRollOff75.0_sma_de_meanPeakDist
pcm_Mag_spectralSkewness_sma_de_minRangeRel
pcm_Mag_spectralSlope_sma_de_peakMeanAbs
pcm_Mag_harmonicity_sma_de_peakRangeAbs
mfcc_sma_de[2]_meanRisingSlope
mfcc_sma_de[7]_meanPeakDist
mfcc_sma_de[7]_meanRisingSlope
mfcc_sma_de[9]_peakDistStddev
mfcc_sma_de[14]_peakRangeAbs
